# Changes in Scleral Thickness Following Repeated Anti-vascular Endothelial Growth Factor Injections

**DOI:** 10.18502/jovr.v17i2.10790

**Published:** 2022-04-29

**Authors:** Yao Wang, FRCSC; Wei Sim, MA; Patrick Wang, Rachel Y. Oh, Timothy Ratzlaff, Jacob Rullo, Sanjay Sharma

**Affiliations:** ^1^Department of Ophthalmology, Queen's University, Kingston, ON, Canada; ^2^School of Medicine, Queen's University, Kingston, ON, Canada

**Keywords:** Intravitreal Injection, Sclera, Macular Degeneration, Macular Edema, Vascular Endothelial Growth Factor

## Abstract

**Purpose:**

This cross-sectional study aimed to compare changes in scleral thickness between eyes injected with repeated anti-vascular endothelial growth factor (anti-VEGF) drugs and fellow injection naive eyes using optical coherence tomography (OCT).

**Methods:**

A total of 79 patients treated with three intravitreal anti-VEGF injections in one eye versus no injections in the fellow eye were included.Anterior segment-OCT measured scleral thickness in the inferotemporal quadrant 4 mm away from the limbus.

**Results:**

Injected eyes had a mean scleral thickness of 588 ± 95 μm versus 618 ± 85 μm in fellow naïve eyes (*P* < 0.001). Comparing injected eyes to fellow naïve eyes stratified by injection number showed a mean scleral thickness of 585 ± 93 μm versus 615 ± 83 μm in eyes with 3–10 injections (*n* = 32, *P* = 0.042); 606 ± 90 μm versus 636 ± 79 μm in eyes with 11–20 injections (*n *= 24, *P* = 0.017); and 573 ± 104 μm versus 604 ± 93 μm in eyes with > 20 injections (*n* = 23, *P* = 0.041). There was no significant correlation between injection number and scleral thickness change (*r* = –0.07, *P* = 0.26). When stratified by indication, subjects with retinal vein occlusions showed a statistically significant difference in scleral thickness between injected and fellow naïve eyes (535 ± 94 μm and 598 ± 101 μm, respectively, *P* = 0.001).

**Conclusion:**

Compared to injection naive eyes,multiple intravitreal injections at the repeated scleral quadrant results in scleral thinning. Consideration of multiple injection sites should be considered to avoid these changes.

##  INTRODUCTION

Anti-vascular endothelial growth factor (anti-VEGF) drugs have revolutionized the treatment of retinal and macular diseases. Conditions treated with anti-VEGF drugs include diabetic macular edema (DME), neovascular age-related macular degeneration (nAMD), and many other causes of macular disorders, with the goals of limiting progression of and/or stabilizing disease, and even reversing visual loss. Various treatment regimens of anti-VEGF drugs have been compared and most are centered on providing injections over the duration of disease activity that can last many years.^[1]^ Injections are administered in an area typically in the superotemporal or inferotemporal quadrant and approximately 3–4 mm posterior to the limbus by penetrating the sclera. It has been hypothesized that numerous injections in a precise location may lead to scleral damage.^[2]^ Recent advances in anterior segment optical coherence tomography (AS-OCT) have allowed for more detailed views of the sclera and surrounding structures, thus enabling more accurate quantification of changes in the scleral architecture.^[3]^

Only one study has been published regarding changes in scleral architecture following intravitreal injections.^[2]^ The authors of this study reported significant scleral thinning of the injected inferotemporal quadrant when retrospectively comparing eyes receiving multiple injections ( ≥ 30, study eye) to the fellow eyes receiving fewer injections ( < 10, control eye). In order to avoid damage to the sclera, it was recommended the injection site be alternated. However, given the control eye still received injections, it is unclear how injected eyes compare to an injection-naïve eye that has not incurred any theoretical changes. To better characterize the effects of intravitreal anti-VEGF injections on the scleral architecture, we compared changes in OCT-measured scleral thickness in eyes receiving intravitreal anti-VEGF compared to fellow eyes of the same patient that did not. This article describes the changes in scleral architecture following repeated intravitreal anti-VEGF injections.

**Table 1:** Summary of clinical data


	**Injected eye (** ***n*** ** = 79)**	**Naïve eye (** ***n*** ** = 79)**
**Number of intravitreal injections**	16.37 ± 13.1	
** ${}^{\#}$ Ranibizumab (Lucentis)**	21	
** ${}^{\#}$ Aflibercept (Eylea)**	12	
** ${}^{\#}$ Bevacizumab (Avastin)**	1	
** ${}^{\#}$ Combination**	45	
**IOP baseline, mmHg**	14.44 ± 2.8	14.36 ± 2.9
***P-*** **value**	0.208	1.000
	
	

**Table 2:** Scleral thickness measurements summary in the injected quadrant


**Injected eye thickness, μ m**	**Naïve eye thickness, μ m**	***P*** **-value**
588 ± 95 ${}^{*}$	618 ± 85 ${}^{*}$	< 0.001 ${}^{*}$
	
	

**Table 3:** Scleral thickness as stratified by number of injections


	**Number of injections (range)**	**Injected eye scleral thickness, μ m**	**Naïve eye scleral thickness, μ m**	***P*** **-value**
**3–10 injections (** ***n*** ** = 32)**	5.1 ± 2 (3–9)	585 ± 93 ${}^{*}$	615 ± 83 ${}^{*}$	0.042 ${}^{*}$
**11–20 injections (** ***n*** ** = 24) **	14.9 ± 3 (12–20)	606 ± 90 ${}^{*}$	636 ± 79 ${}^{*}$	0.017 ${}^{*}$
** > 20 injections (** ***n*** ** = 23)**	33.7 ± 10 (21–60)	573 ± 104 ${}^{*}$	604 ± 93 ${}^{*}$	0.041 ${}^{*}$
	
	

**Table 4:** Scleral thickness and IOP measurements as stratified by indication


	**Naïve eye thickness, μ m**	**Injected eye thickness, μ m**	***P*** **-value**
**AMD **	629 ± 84 (*n* = 39)	606 ± 97 (*n* = 39)	0.073
**DME **	611 ± 69 (*n* = 20)	592 ± 71 (*n* = 20)	0.090
**RVO **	598 ± 101 ${}^{*}$ (*n* = 17)	535 ± 94 ${}^{*}$ (*n* = 17)	0.001 ${}^{*}$
**Other **	640 ± 118 (*n* = 3)	624 ± 144 (*n* = 3)	0.656
	
	

**Figure 1:**
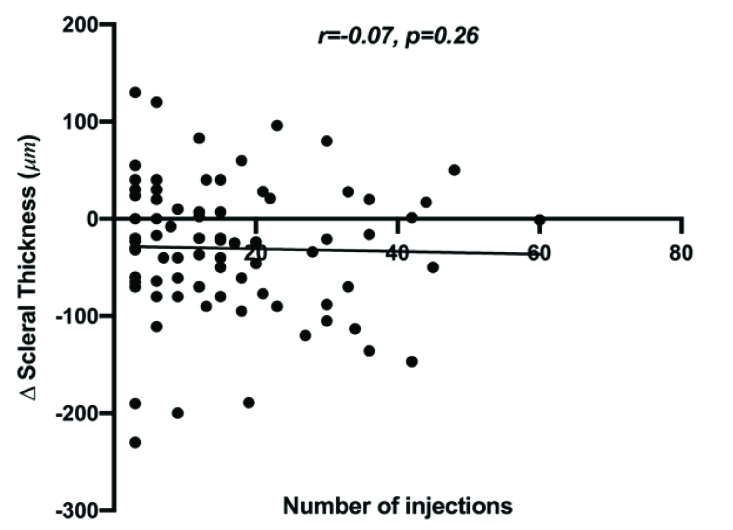
Correlation of number of injections with scleral thickness change in injected and injection-naïve fellow eye ( Δ = difference, *n* = 79, Spearman *r *= –0.072, *P* = 0.263).

**Figure 2:**
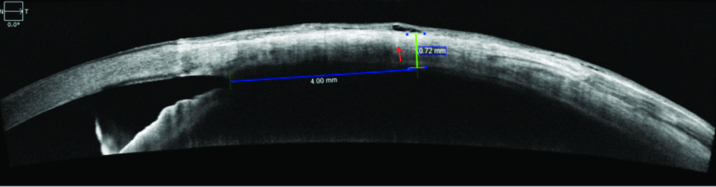
Anterior segment OCT demonstrating changes of conjunctiva after multiple injections. Episcleral cystoid cavity is displayed (*green* *arrow) *corresponding well with the injection site marked by the injection tract (*red arrow).*

##  METHODS

### Selection and Description of Participants

Patients receiving intravitreal injections for various retinal and macular diseases in one eye only, with no previous injections in the fellow eye were considered for inclusion in this cross-sectional study. Injections of 0.05-mL anti-VEGF administered monthly three times or more to the eye diagnosed with a condition indicated for injection were used. Further injections were given based on disease activity noted on OCT and/or clinical exam. All injections were administered to the inferotemporal quadrant at a distance of 3–4 mm from the limbus depending on the patient's phakic status (this was 4 mm for phakic eyes and 3 mm for pseudophakic eyes).

Patients who met the following criteria were included: age 18 or over and those receiving ≥ 3 anti-VEGF injections in only one eye for retinal diseases including nAMD, DME, and RVOs. Patients with coexisting conditions that might skew scleral thickness such as filtrating glaucoma surgery and scleral buckles, and/or poor-quality scleral OCT images unsuitable for measurement were excluded.

### OCT Imaging and Analysis 

A spectral domain OCT with an anterior segment module was used to image the sclera. To ensure a high-quality image of the scleral structures, the site of injection in the inferotemporal quadrant 3–4 mm from the limbus in each injected and injection-naïve eye was imaged by having the patient fixate on a light. Scleral thickness was measured with an integrated caliper tool on the OCT by a masked observer capable of identifying anterior segment anatomy. Important structures like the episcleral vascular plexus and injection tracts were identified to help distinguish the sclera from the overlying conjunctiva and localize the injection site.

### IOP Measurements

Intraocular pressure (IOP) was measured using the Goldman applanation tonometer at baseline in both eyes before any injections.

### Statistical Analysis 

Collected data were stored and statistically analyzed on Prism 8 (GraphPad Software Inc., CA, USA). Corresponding means and standard deviations were used to express continuous quantitative variables. d'Agostino and Pearson omnibus normality testing were used to check for normal distribution of data. Paired two-tailed *t*-tests were used to assess scleral thickness changes within groups. Correlation between changes in scleral thickness and number of injections was determined using Spearman. A *P*-value ≤ 0.05 was considered as statistically significant.

##  RESULTS

Of the recruited patients, 79 had successful scleral images with quality suitable for measurement. The mean age was 73 years (range, 34–94) with 49 females and 30 males. Indications for anti-VEGF injection were as follows: 17 patients with RVOs, 39 with nAMD, 20 with DME, and 3 with other causes (i.e., histoplasmosis, central serous chorioretinopathy). Summarized in Table 1 are the medications used, total number of injections, and baseline IOP measurements.

Injected eyes had a mean inferotemporal quadrant scleral thickness of 588 ± 95 μm versus 618 ± 85 μm in non-injected eyes (*P* < 0.001) [Table 2]. Stratifying by the number of injections, the mean scleral thickness of 606 ± 90 μm in the eyes with 11–20 injections (*n* = 24) versus 636 ± 79 μm in the injection-naïve fellow eyes (*P* = 0.017); 585 ± 93 μm in the eyes with 3–10 injections (*n* = 32) versus 615 ± 83 μm in the injection-naïve fellow eyes (*P* = 0.042); and 573 ± 104 μm in the eyes with > 20 injections (*n* = 23) versus 604 ± 93 μm in the injection-naïve fellow eyes (*P* = 0.041) [Table 3]. Furthermore, between the injected and injection-naïve fellow eyes in the RVO group, there was a significant difference in scleral thickness when stratified by indication (535 ± 94 μm and 598 ± 101 μm, respectively; *P* = 0.001) [Table 4].

Clearly visible injection site marks were noted on several OCT images [Figure 2]. Many patients exhibited episcleral cystoid cavities seen on OCT in the eye receiving intravitreal injections. Analyzing the correlation between the number of injections and change in scleral thickness found a Spearman correlation of –0.07 (*P* = 0.26) implying that the degree of change in scleral thickness did not correspond with a higher number of injections [Figure 1].

##  DISCUSSION

Sclera is an important structural element in protecting the globe from external forces and injury. In humans, the sclera is mostly composed of collagen types I, II, V, and VI, proteoglycans, and water.^[4]^ Reported scleral thickness measurements in enucleated specimens have yielded on average 430 nm at the ora serrata while others using ultrasound have found an approximate thickness of 550 nm at 3 mm from the limbus.^[5,6]^ Our measurements are slightly higher than the reported which could be due to technical differences in the method used to measure the scleral thickness.

Recent advances in OCT technology with enhanced depth imaging have allowed detailed visualization of the sclera. Sclera is used as an entry point to deliver medications into the eye. As performing multiple intravitreal injections becomes widespread, it raises the question whether repeated trauma to the sclera ultimately changes its architecture. We have shown that multiple intravitreal injections result in localized thinning of the sclera relative to the injection-naïve eye.

Few theories about the mechanism of thinning have been postulated with regards to the changes observed. The first is simply mechanical stress. Scar tissue formed in the injection path may result in fibrosis and impaction of collagen. Second, anti-VEGF antibodies may have a direct effect on scleral hydration. Reflux through the injection site may lead to concentrated levels of anti-VEGF.^[7]^ Collection of anti-VEGF in these focal pockets may alter scleral vessel permeability and cause dehydration or localized thinning after recurrent injections. Reflux can also cause the vitreous to become trapped in the injection path which has been reported with vitreoretinal surgeries, further contributing to the thinning process. Third, anti-VEGF injections associated with elevated IOP may be another etiological factor as studies of experimentally induced glaucoma or in animal models have demonstrated significant scleral thinning.^[8,9]^

One study found that thinning of the sclera was most prominent at IOP levels of 60 mmHg or higher, possibly leading to thinning of nearby inferonasal and superotemporal quadrants.^[10]^ Our data did not show any significant differences in IOP in the injected and naïve eyes [Table 1].

Interestingly, correlation between thickness and the number of injections was not found, implying that although scleral thinning occurs initially with intravitreal injections, further thinning does not occur with increased number of injections [Figure 1]. This could be explained by the phenomenon of stiffening of the sclera that occurs when exposed to mechanical strain and acute elevations of IOP (which have been reported to be extremely common after anti-VEGF injections).^[11,12,13]^ Several studies have shown that scleral fibroblasts activate the release of matrix metalloproteinases when subjected to mechanical strain, ultimately resulting in remodeling of the extracellular matrix.^[13,14]^ The sclera's biomechanical response is a protective mechanism that minimizes deformations and future remodeling in response to increased IOP.^[12]^

A significant difference in scleral thickness between the injected and fellow injection-naïve eyes was seen only in the RVO subgroup. One prior study evaluating scleral thickness in subjects with central RVO found measurements at and around (1, 2, and 3 mm from) the sceral spur to be significantly thicker than healthy controls. Thus, significant thinning following intravitreal anti-VEGF injections in RVO patients may be possibly due to inherent pathological changes of the sclera predisposing them to deformation, given this difference was not seen in the nAMD and DME subgroups.

Limitations of this study are due to its relatively small sample size and cross-sectional nature. To accurately assess the temporal relationship of intravitreal injection on scleral thickness, measurements of the sclera in the same eye before injection would ideally be used as controls. While our study used the contralateral eye as a control, we attempted to reduce confounding by only including patients who did not have coexisting ocular disease or previous intravitreal injections in the contralateral eye. Hypothetically, anatomical differences between eyes would be expected to be minimal but does not eliminate the possibility for variation between eyes. Although scleral thickness measurements were performed by a trained assessor, human error and variability in measurements may exist. For future studies, we would consider making multiple scleral thickness measurements to obtain the error associated with the measurements (mean +/– SE) from each image and have multiple individuals obtaining measurements from each image to find a mean thickness measurement. Additionally, reading images could be performed in a random order independently to minimize intraobserver variability. Acute imaging showing the injection tract obtained immediately following injections may ensure increased accuracy in the measured location. Comparison of measured scleral thickness in all four quadrants and axial length between injected and injection-naïve eyes may eliminate other factors contributing to observed differences such as hyperopes and emmetropes possessing increased scleral stiffness versus myopes.^[15]^ A prospective study measuring scleral thickness before and after the onset of intravitreal injections to confirm temporal changes over time is most ideal.

To the authors' best knowledge, this is the first study to evaluate the effect of repeated anti-VEGF intravitreal injections on the sclera when comparing injected eyes to injection-naïve eyes of the same patient. As a result of scleral thinning that occurs, alternating intravitreal injection sites may curb these observed changes.

##  Financial Support and Sponsorship

The authors received no financial support for the research, authorship, and/or publication of this article.

##  Conflicts of Interest

The author(s) declare no potential conflicts of interest with respect to the research, authorship, and/or publication of this article.
